# Clinical Utility of the DriveFocus™ Intervention on Young Drivers With and Without Experience

**DOI:** 10.3389/fpubh.2019.00123

**Published:** 2019-05-24

**Authors:** Liliana Alvarez, Sherrilene Classen, Shabnam Medhizadah

**Affiliations:** ^1^School of Occupational Therapy, Faculty of Health Sciences, University of Western Ontario, London, ON, Canada; ^2^Department of Occupational Therapy, College of Public Health and Health Professions, University of Florida, Gainesville, FL, United States

**Keywords:** adolescents, young drivers, driving simulation, hazard perception, DriveFocus™

## Abstract

**Introduction:** The DriveFocus™ intervention addresses the ability of young drivers to detect and respond to critical roadway information. DriveFocus is an interactive video-based tablet application that teaches users how to detect and prioritize critical roadway items. However, young drivers with and without experience may respond differently to the intervention.

**Objectives:** We compared the simulated driving performance of two 17 year-old licensed drivers with (novice) and without (learner) driving experience, after three (post-test 1) and six (post-test 2) intervention sessions.

**Methods:** We collected clinical, driving performance (number of visual scanning, adjustment to stimuli, and total driving errors) and acceptability data.

**Results:** The learner driver made more visual scanning, adjustment to stimuli and total errors when compared to the novice. Both participants exhibited a decrease in both types and number of driving errors from baseline to post-test 2 and the learner also made less driving errors at post-test 1. Both participants rated the perceived ease of use of the intervention favorably.

**Conclusions**: This study lays the foundation to examine the impact of the DriveFocus™ intervention among novice and more experienced young drivers.

## Introduction

Motor vehicle collisions (MVCs) are the leading cause of death among youth worldwide (1). In Canada, youth between 15 and 24 years account for over 20% of road traffic fatalities and serious injuries. This is particularly concerning when considering they represent only 13% of the driving population (2). The overrepresentation of youth in MVC statistics, a global public health concern, is the result of numerous factors. Brain maturation, the lack of driving experience, the influence of peer passengers, overconfidence in their driving skills, and decreased ability to detect (visual scanning) and respond (adjustment to stimuli) to and sustain attention to critical roadway information are among the prevailing characteristics of youth driving (3, 4). Together, these factors place young drivers at a high-risk for distracted driving.

Distracted driving occurs when attention shifts away from the driving task toward an un-related object or activity (5). Driving distraction can occur when the driver takes the eyes off the road (visual distraction), the mind off the task (cognitive distraction), and/or the hands off the wheel (manual distraction) (6). Driving is a demanding task that requires the integration of motor, cognitive, and visual-perceptual skills in order to operate the vehicle while detecting and responding to hazards and roadway information. Thus, distraction severely compromises fitness to drive, or the ability to control a motor vehicle smoothly and cautiously, while keeping up with the flow of traffic (7).

Every year, distracted driving causes approximately 4 million MVCs in North America (8). Moreover, 58% of MVCs in the teen population involve some form of distraction (9). Novice drivers in their first 3 years of driving experience, a large proportion of whom are young drivers, are at an increased crash risk for distracted driving compared to experienced drivers (10, 11, 46). This is concerning given that driving distraction can result even when the driver is not physically engaged in a secondary task (e.g., texting, reaching for a phone, eating, etc.). Distracted drivers may look at -and yet fail to attend to almost 50% of the roadway information in their driving environment (12). Currently available interventions for young drivers focus on increasing risk-awareness, law enforcement, and ensuring extended and supervised learning experience through graduated licensing programs (13). However, a need remains for intervention strategies that can improve the underlying ability of young drivers to attend and respond to critical roadway information (14). Furthermore, although experience and age are moderators of distracted driving in young drivers, they can have independent effects on driving performance. As such, interventions for this population must elucidate any differential effects on novice drivers with and without experience (15).

This descriptive case study compares the utility of the DriveFocus® app-based intervention on the simulated driving performance of two 17 year-old male drivers with and without driving experience based on the different stages of their graduated licensing process. This fidelity (16) and feasibility tested intervention (42) shows potential to reduce the type and number of errors made by youth between the ages of 16 to 24 in a pilot driving simulator study (43). As such, this case study lays the foundation for investigating the efficacy of the intervention while accounting for any potential effects on young drivers with differing levels of experience. Such knowledge is necessary in order to design targeted intervention strategies to address the specific needs of young novice drivers.

## Methods

We compared the simulated driving performance of two 17 year-old licensed drivers with three years of driving experience (hereon referred to as “novice”) and without driving experience (hereon referred to as “learner”). The learner and novice drivers participated in the DriveFocus™ intervention, an interactive video-based intervention that teaches young drivers how to detect and respond to roadway information, delivered via a tablet application. The participants' driving performance was assessed before and after three and six intervention sessions via a DriveSafety CDS-200 high fidelity driving simulator (DriveSafety, Salt Lake City, Utah). We included participants if they: (a) were between 16 and 19 years of age; (b) had a valid G1 or G2 learner's permit according to the province's graduated licensing program (a G1 license allows drivers with no prior experience to drive only when accompanied by a fully licensed adult, and a G2 allows the driver with at least 1 year of prior experience to drive independently with certain restrictions after having passed the province's on-road test); (c) were proficient in English; and (d) were able to travel to the research laboratory. Participants were excluded if they: (a) had experienced a seizure in the previous year; (b) had a physical or mental health diagnosis that would preclude participation; (c) were taking any medications that could negatively impact their driving performance; (d) did not meet the provincial visual acuity requirement for driving (20/50 with both eyes opened and examined together); or (e) did not meet the provincial visual field requirement for driving (120° horizontal continuous visual field) (17). Participants received a $25 movie theater gift card and a $20 gas voucher for their participation in this study.

The University of Western Ontario's Nonmedical Research Ethics Board approved this study (#107267). Both participants and their parent/legal guardian provided written informed consent to participate in this study and agreed to the publication of these results without the use of their names or personal health information.

## Participants

The learner driver was a 17-year old Caucasian college student living on-campus, with a G1 driver's license and no prior driving experience. The learner driver had obtained his G1 license a year prior to enrollment in the study and had not driven prior to or after obtaining his license.

The novice driver was also a 17-year old Caucasian male and college student, with a G2 valid driver's license with 3 years of driving experience, including 1 year of experience with a G1 license under supervision. He had no history of prior collisions or driving citations. He had recently moved to live on-campus where he did not own a vehicle and was not driving at the time. The latter was the reason for not having pursued his full license. Although this driver had prior experience, he still had not graduated to a full license which requires demonstrated competency in highway or interstate driving, and thus is referred to as a novice driver throughout the study.

## Procedure

All assessment and intervention sessions were conducted at the University of Western Ontario, Canada. Participants completed a baseline assessment followed by three intervention sessions, post-test 1, three additional intervention sessions and post-test 2. The baseline assessment consisted of: a demographic questionnaire, a battery of clinical assessments (Table 1), orientation to the simulator and 7 min acclimation drive, and one of three 15 min main drives. Main drives were randomly assigned to each participant at baseline, post-test 1 and 2, and conducted in the CDS-200 high fidelity simulator. Post-test 1 consisted only of the simulator acclimation and main drives, and post-test 2 included simulator acclimation and main drives, as well as clinical assessments.

**Table 1:** Summary of clinical assessments results for two young drivers (with and without driving experience) at baseline.

**Clinical assessment**	**Scoring**	**Results for P1 (no driving experience)**	**Results for P2 (three years of driving experience)**
**Vision (OPTEC 2500 visual analyzer)**			
Visual Acuity for both eyes, right eye, left eye)	Provincial requirement is ≤ 20/50 with both eyes examined together	Intact binocular 20/20 Intact R eye 20/20 Intact L eye 20/20	Intact binocular 20/20 Intact R eye 20/20 Intact L eye 20/20
Peripheral field of view	Provincial requirement is ≤ 120° continuous with both eyes examined together	Intact 170°	Intact 170°
Color discrimination	Intact: Correctly identified 6 ≤ items Impaired: ≤ 5	Intact 7/8	Intact 8/8
Depth perception	Intact: Correctly identified 5 ≤ items Impaired: ≤ 4	Intact 8/8	Intact 6/8
Lateral phorias	Intact: Binocular fixation between 4 and 13 Impaired: ≤ 3 or ≥ 14	Intact (9)	Intact (10)
Vertical phorias	Intact: Binocular fixation between 3 and 5 Impaired: ≤ 2 or ≥ 6	Intact (5)	Intact (4)
Contrast sensitivity	Intact: The curve is within normal range of the record chart Impaired: Any point in the curve is outside the normal range	Intact	Intact
**Visual-motor integration**			
Beery VMI	30/30 points indicates adequate age-related visual-motor integration skills	Intact 29/30 at the 61^st^ percentile	Intact 29/30 at the 61^st^ percentile
Cognition			
CTMT	Time in seconds to complete each of the five sequencing trails	Intact at 89th percentile Trail 1 = 22 s Trail 2 = 21 s Trail 3 = 33 s Trail 4 = 16 s Trail 5 = 40 s Sum of T scores = 307	**Impaired at 10th percentile** Trail 1 = 37 s Trail 2 = 46 s Trail 3 = 48 s Trail 4 = 35 s Trail 5 = 39 s Sum of T-scores = 195
SDMT	Number of correctly identified symbol correspondence in 90 s. Highest possible score = 110	Average Completed 69/110 symbols Correctly identified 68/69	Average Completed 59/110 symbols Correctly identified 55/59
**Motor skills**			
BOT-2 Short Form	Points for each of the 8 motor skill sub-tests. Highest possible score = 88 points	Average Percentile rank = 35th Score = 74	Average Percentile rank = 35th Score = 75
TCI-R	Profiles the participant according to four temperament traits (novelty seeking; harm avoidance; reward dependence; persistence) and three character traits (self-directedness; cooperativeness; self-transcendence)	NS = 5.7% **HA=** **18.1%** RD = 9% P = 7.9% SD = 19.9% **Coop=** **24.3%** ST = 15%	NS = 6.4% HA = 9.8% RD = 12% **P=** **13.9% SD=** **21.2%** Coop = 20.8% ST = 16.3%

*Beery VMI, Beery-Buktenica Developmental test of Visual Motor Integration; CTMT, Comprehensive Trail making Test; SDMT, Symbol Digit Modality Test; BOT-2, Bruininks-Oseretsky test of Motor Performance; TCI-R, Revised Temperament and Character Inventory; Ns, Novelty Seeking; HA, Harm Avoidance; RD, Reward Dependence; P, Persistence; SD, Self-directedness; Coop, Cooperativeness; ST, Self-transcendence. Bolded items indicate differences between participants*.

## Intervention

The DriveFocus™ app consists of six sets of interactive drives through different North American cities, that aim to teach users how to detect and prioritize critical roadway items (e.g., a pedestrian crossing in front of the vehicle is prioritized over the traffic light). Critical items include pedestrians, bicyclists, the brake lights of the lead vehicle, traffic lights, stop signs, road markers, construction signs, and speed and regulatory signs. Through each interactive video drive the participant clicks on these critical roadway items as they appear. When an item is correctly identified and prioritized, the app provides an auditory and visual cue to indicate a correct response. After each drive, the app provides the participant with a results summary including number of correctly identified items, missed items, and associated reaction times. Participants completed one set per session for a total of six intervention sessions.

## Measures

### Battery of Clinical Assessments

Participants completed a validated battery (Table 1) of clinical assessments appropriate for this population (18). The OPTEC 2500 visual analyzer, a sensitive and specific screening tool for visual health (19) was used to assess seven visual skills: visual acuity, peripheral field of view, color discrimination, depth perception, lateral phorias, vertical phorias, and contrast sensitivity. Visual-motor integration was measured via the Beery-Buktenica Developmental Test of Visual-Motor Integration [Beery VMI™; (20)], a valid and reliable norm referenced test of visual motor integration (20). Cognitive skills were assessed through the Comprehensive Trail-making Test [CTMT; (21)], a standardized and valid measure of mental flexibility and set-shifting (41); and the Symbol Digit Modalities Test [SDMT; (22)], a reliable and valid test of divided attention, visual scanning, and tracking (23). We also measured gross and fine motor skills via the standardized and norm-referenced Bruininks–Oseretsky Test of Motor Proficiency [BOT-2; (24, 25)]. Finally, participants completed the Revised Temperament and Character Inventory [TCI-R; (26)], a standardized personality test that profiles the participant according to four temperament (novelty seeking, harm avoidance, reward dependence, and persistent) and character (self-directedness, cooperativeness, and self-transcendence) traits.

### Driving Performance

To prevent the onset of simulator adaptation syndrome, participants followed standard protocol (27) and drove an acclimation scenario. Simulator adaptation syndrome refers to a group of symptoms that drivers might experience when exposed to a driving simulator scenario, including dizziness, light-headedness, queasiness, and/or nausea. Such symptoms can be mitigated by implement evidence-based strategies including acclimation scenarios. This scenario included a straight drive with minimal visual activity and constant speed, followed by a straight drive requiring brief stops at stop signs. The driver was then exposed to a route requiring four left turns, followed by four right turns. In these routes all road users abide by the rules of the road and no critical events are simulated. Participants were then exposed to the main drives which started in a residential area and progressed to a downtown area. Highway driving was not included as drivers in the graduated licensing program have restrictions that are specific to interstate conditions. Main drives included five hazardous events presented in different order and designed to challenge participants' visual scanning and adjustment to stimuli abilities: (1) a pedestrian unexpectedly crossing in front of the vehicle; (2) a car making a sudden lane change; 3) a vehicle coming out of the parking lane; (4) a sudden change of traffic light upon arrival to an intersection; and (5) and a strategic navigation task requiring participants to use sign postings to make the correct turn toward a pre-set destination (i.e., Toronto or London, Ontario). All drives were recorded with a dual camera view point (Exmor R™, Sony), capturing the visual field of the driver as well as their eye gaze. Once each participant had completed baseline, post-test 1 and 2 each drive was independently evaluated by an occupational therapist. The occupational therapist used a scoring sheet specifically customized to the characteristics of the route to record the number of visual scanning, adjustment to stimuli and total errors made by each participant. The occupational therapist was blinded to the clinical outcomes of each participant, as well as the status of the drive (i.e., pre or post-test), and the participant's driving experience.

### Acceptability of the Intervention

Participants completed the Perceived Ease of Use scale [PEOU; (28)] adapted for this study. Perceived ease of use shapes a person's attitudes toward and intention to use a technology (29), and therefore constitutes a critical determinant of technology acceptance. The scale consists of six statements regarding the technology and the participant is asked to rate each statement on a 7-point scale from extremely unlikely to extremely likely. In addition, participants were asked to use a 5-point scale (from 1 = strongly disagree to 5 = strongly agree) in response to the additional item “I think I would like to use this app frequently.” Finally, participants were asked to indicate which of the following they believed was most helpful for their driving, if at all: the app intervention, the assessment simulator drives, or both.

## Results

### Battery of Clinical Assessments

Both participants had age-appropriate visual, motor and cognitive skills (Table 1), with the exception of the CTMT where the novice driver performed at the 10th percentile with a T-scores sum of 195 (at the 51th percentile the T-scores sum for the age range is 250–254). There were no differences in visual, visual-motor integration, and motor skills between the two participants. In the TCI-R, the learner's profile was characterized by a greater influence of harm avoidance temperament and cooperativeness trait, while the novice's profile indicated a greater persistence temperament and self-directedness character.

### Driving Performance

**Figure 1:**
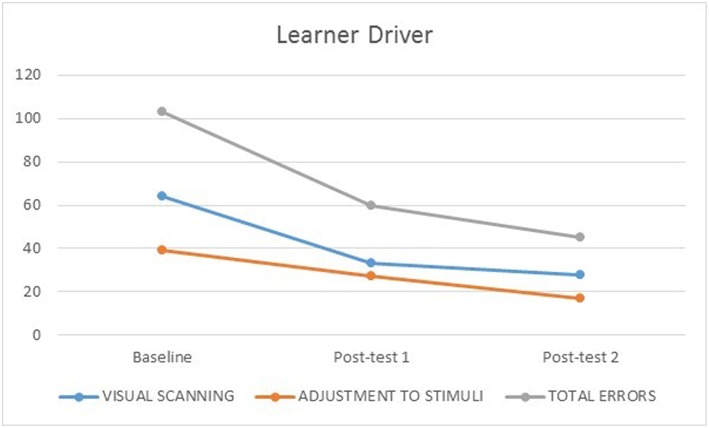
Learner driver's number of visual scanning, adjustment to stimuli, and total errors at baseline, post-test 1 and 2.

**Figure 2:**
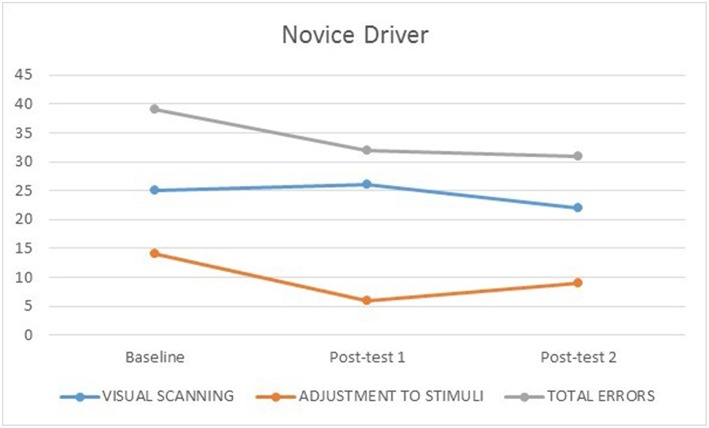
Novice driver's number of visual scanning, adjustment to stimuli, and total errors at baseline, post-test 1 and 2.

None of the participants experienced simulator adaptation syndrome. Figures 1, 2 illustrate the number of visual scanning, adjustment to stimuli, and total driving errors for both participants across baseline, post-test 1 and post-test 2. The learner made more visual scanning, adjustment to stimuli and total driving errors at baseline, post-test 1 and post-test 2 compared to the novice driver who had driving experience. The learner made less errors at post-test 1 compared to baseline, and both the learner (visual scanning errors at baseline = 64; post-test 1 = 33; post-test 2 = 28) and novice (visual scanning errors at baseline = 25; post-test 1 = 26; post-test 2 = 22) drivers made less visual scanning errors at post-test 2 compared to baseline.

The learner made less adjustment to stimuli errors from baseline (39) to post-test 1 (27) and 2 (17); while the novice driver made few adjustment to stimuli errors across baseline (14), post-test 1 (6) and post-test 2 (9), which may be consistent with his increased driving experience. Both the learner (baseline = 103, post-test 1 = 60, 2 = 45) and novice (baseline = 39, post-test 1 = 32, 2 = 31) drivers made less total driving errors from baseline to post-test 1, and post-test 2.

### Acceptability of the Intervention

Table 2 shows the PEOU scores for both participants. Both the learner and novice drivers assigned the app very favorable ratings (between 6 and 7) for items 1, 3, 5, and 6. For item 2 (“I found it easy to get the DriveFocus™ app to do what I wanted it to do”) both participants assigned moderately favorable ratings (4 or 5). For item 4 (“I found the DriveFocus™ app to be flexible to interact with”), the learner assigned a moderately favorable rating (5) while the novice driver assigned a very favorable rating (6). Both participants indicated a neutral rating (“neither unlikely nor likely”) on the item “I think I would like to use this app frequently,” and both found the app to be most helpful for their driving skills.

**Table 2:** Perceived Ease of Use scale and acceptability items for P1 and P2.

**Item**	**Statement**	**P1**	**P2**
1	Learning to operate the DriveFocus app was easy for me	6	6
2	I found it easy to get the DriveFocus app to do what I wanted it to do	4	5
3	The DriveFocus app was clear and understandable	6	6
4	I found the DriveFocus app to be flexible to interact with	5	6
5	It was easy for me to become skillful at using the DriveFocus app	7	6
6	I found the DriveFocus app easy to use	6	6
7	I think I would like to use this app frequently	4	4
8	The following was most helpful for my driving, if at all:	app	app

*Participants rated each item in a 7-point scale where: 1, Extremely unlikely; 2, Quite unlikely; 3, Slightly unlikely; 4, Neither unlikely nor likely; 5, Slightly likely; 6, Quite likely; 7, Extremely likely. Items 1 to 6 were rated on a scale from 1 to 7. Item 7 was rated on a scale from 1 to 5*.

## Discussion

The aim of this study was to illustrate the differences in driving performance between two young drivers with and without experience, in response to six sessions of the DriveFocus™ intervention. Both participants had similar and age-appropriate visual, visual-motor, cognitive and motor skills, with the exception of the novice driver who performed at the 10th percentile in the CTMT. Performance in the CTMT has been associated with driving outcomes in older adults with (30) and without impairments (31). However, less is known about the predictive validity of the CTMT on adolescent driving performance. Recent studies report a correlation between CTMT scores and overall executive functioning, an important contributor to driving outcomes in this population (32). Nevertheless, the heterogeneity of executive function measures has limited the understanding of performance-based measures and driving outcomes (32). Although future research may help illuminate the impact of low CTMT scores in relation to driving performance, it is well-established that obtaining one low score among a full battery of clinical tests is common among adolescents (33). Thus, given the age-appropriate performance of the novice driver across all other tests, this was not interpreted as a concern. Both participants had different temperament and character profiles according to the TCI-R, yet neither was characterized as novelty seeking, a temperament trait that is correlated with risk-taking behaviors (34). Although researchers (35–37) examined personality traits on the fitness to drive abilities of middle-age and older drivers, to our knowledge, no study to date has examined the relationship between temperament and character traits in the TCI-R and driving outcomes, in the youth population. Future research is needed with adequately powered samples to identify any existing correlations.

The learner driver made more visual scanning, adjustment to stimuli and total errors when compared to the novice driver, which may be consistent with positive effects of experience on the driving performance of young drivers (38). Both participants seemingly benefited from the intervention. The learner driver experienced a 48.5% decrease in the number of visual scanning errors after three intervention sessions (post-test 1) and 56.25% after six sessions. Similarly, the learner driver showed a decrease in the number of adjustment to stimuli errors from baseline to post-test 1 (30.8%) and post-test 2 (56.4%). The novice driver, on the other hand, exhibited a slight decrease in the number of visual scanning errors from baseline to post-test 2 (12%) but not post-test 1; as well as the number of adjustment to stimuli errors from baseline to post-test 1 (57.1%) with a slight increase from post-test 1 to post-test 2 (35.7% when compared to baseline). These results indicate that the participant with no prior driving experience benefited from the intervention to a greater extent than the participant with prior experience. These results need to be empirically validated with a larger sample in which effect sizes can be quantified. However, if confirmed, they can assist occupational therapists, driving rehabilitation specialists and driving school instructors in the use of this app-based intervention with young learner and novice drivers.

Finally, participants indicated overall perceived ease of use of the DriveFocus™ app, with highly favorable ratings. Item 2, “I found it easy to get the DriveFocus app to do what I wanted it to do” was rated as moderately favorable as both participants experienced slowed downloading and refreshing times at some point during their intervention sessions. Resolving such issues in future versions and ensuring release updates may help overcome these challenges. Although both participants were new to driving simulation, they perceived the app to be more helpful than the actual simulator, for their driving skills. This indicates that young drivers with and without experience may find the app an acceptable and suitable intervention for practicing driving skills, a characteristic that increases adherence (39).

Recent literature on distracted driving highlights the need for evidence-based strategies and interventions directed at changing the behavior of the driver and reshaping their habits (44, 45). Moreover, people have prevalent misperceptions around their ability to multitask and the extent to which they are vulnerable to risk and missing critical information (40). As such, interventions that can increase awareness while addressing the underlying skills and habits of drivers are imperative. This study lays the foundation for the investigation of an evidence-based intervention that focuses on improving the ability of novice and learner drivers to detect and respond to critical roadway information, while increasing their awareness of such items and their priory status.

## Limitations

The results of this descriptive case study cannot be generalized to the adolescent driver population. Thus, the interpretation of the results warrants caution as it is descriptive of the performance of two specific participants, in Ontario Canada. Furthermore, one of our participants performed below the norm on the CTMT. Although evidence suggests that one low score among a full battery of tests might be typical for adolescents (33), this result might indicate an unreported impairment. This case study may, however, inform the practice reasoning of occupational therapists, driving rehabilitation specialists, and driving school instructors as they partner with young clients in improving their driving skills.

## Conclusion

Young drivers are a high-risk population for motor vehicle collisions. In spite of being highly preventable, MVCs continue to be the leading cause of death for youth between the ages of 15 to 29 across the globe (1). Thus, a need remains for interventions that can improve the underlying skills of young drivers. Such interventions, however, may have the potential to be specifically targeted to the level of experience of the driver or the timing within the graduated licensing program to maximize effectiveness. This descriptive case study illustrates the effects of an app-based intervention on the visual scanning and adjustment to stimuli abilities of two 17-year old drivers with and without experience. By doing so, this case study lays the foundation to compare the utility of the intervention among experienced and novice young drivers. In addition, this study examined the acceptability and suitability of the interventions among the two participants, a critical step in the adherence success of effective interventions.

## Ethics Statement

This study was approved by the University of Western Ontario, Non-medical Research Ethics Board (#107267). The parent study was also registered as an intervention trial in ISRCTN (study ID: ISRCTN66950576). All participants provided informed consent prior to enrolling in the study, in accordance with the Declaration of Helsinki. The parent or legal guardian also provided informed consent. Participants received a $25 movie theater gift card and a $20 gas voucher for their participation in the study.

## Author Contributions

SC encouraged LA to investigate the differences between a driver with and without experience and supervised the findings of this work. LA and SC conceived and conceptualized the case study. LA and SM collected the data and developed the data base. LA performed the analysis. SC verified the analytical methods. All authors discussed the results and contributed to the final manuscript.

### Conflict of Interest Statement

The authors wish to declare that the SC has a professional relationship (previous grants, publications and presentations) with one of the developers of the app. However, no members of the research team (including all authors and research assistants) have any financial interest in the funding company, the app, or any related products.
